# The impact of statin therapy on the healing of diabetic foot ulcers: a case–control series

**DOI:** 10.1186/s40842-024-00175-8

**Published:** 2024-07-10

**Authors:** Brennen O’Dell, Gary Rothenberg, Crystal Holmes, Sari Priesand, Kara Mizokami-Stout, Eric J. Brandt, Brian M. Schmidt

**Affiliations:** 1 Department of Internal Medicine, Division of Metabolism, Endocrinology, and Diabetes, University of Michigan Medical School, Ann Arbor, MI 48106 USA; 2 Department of Internal Medicine, Division of Cardiovascular Medicine, University of Michigan Medical School, Ann Arbor, MI USA; 3 Institute for Healthcare Policy, University of Michigan Medical School, Ann Arbor, MI USA

**Keywords:** Statin, Wound healing, Diabetic foot ulcers, Diabetes mellitus, Hyperlipidemia

## Abstract

**Background:**

Diabetic foot ulcers (DFU) are a costly complication of diabetes mellitus (DM), with significant implications for the patient and the healthcare professionals that treat them. The primary objective of this study was to evaluate if there were improved healing rates in patients with a DFU that were taking a statin medication compared to those patients with a DFU who were not taking a statin medication. Secondary outcomes assessed were correlations with wound healing or statin use on data obtained from retrospective chart review.

**Methods:**

A case–control series was performed to obtain appropriate demographic information, comorbid conditions, laboratory values, and physical examination findings. From the time of presentation with DFU, these patients were followed for 12 weeks to evaluate for healing. Healing was defined as full epithelialization of the DFU with no further drainage. Wound healing and statin use correlation testing was then done for collected variables and each cohort. Chi square and Pearson correlation were then performed to identify any significant correlations. All p-values were two-sided, and findings were considered statistically significant at *p* < 0.05.

**Results:**

Our study identified 109 patients, 75 patients with a DFU on statin medication and 34 patients with a DFU not on statin medication. The statin cohort was more likely to be older, less than 5-year duration of diabetes, have more comorbidities, decreased low-density lipoprotein (LDL) cholesterol, and decreased total cholesterol (*p* < 0.05). Among those patients taking a statin medication, 48.0% (36/75) healed their DFU within 12 weeks. Among those patients not taking a statin medication, 44.1% (15/34) healed their DFU within 12 weeks. No correlation was noted between wound healing and statin use (*p* = 0.7). For wound healing, a negative correlation was noted for prior minor amputations (*p* < 0.05). For statin use, correlations were noted for age, duration of DM, LDL cholesterol level, total cholesterol level, HTN, CAD, and HLD (*p* < 0.05).

**Conclusions:**

Statin medication use did not influence DFU healing rates between cohorts. There was a correlation noted between wound healing and prior minor amputations and between statin use and age, duration of DM, LDL cholesterol, total cholesterol, HTN, CAD and HLD. Additionally, we observed no correlation between DFU healing rates and use of a statin medication.

**Supplementary Information:**

The online version contains supplementary material available at 10.1186/s40842-024-00175-8.

## Background

In 2018, 34.2 million Americans were living with diabetes mellitus (DM) [1]. A patient with a new diabetic foot ulcer (DFU) has a 5% mortality rate within the first year after receiving treatment [2]. At 5 years, that mortality rate increases to approximately 42% [2]. Approximately 16% of newly diagnosed DFU will progress to major lower extremity amputation [3]. Those persons who progress to amputations often have a high metabolic comorbid burden, including dyslipidemia [1].

Approximately 43.5% of DM patients have a non-high-density lipoprotein (HDL) cholesterol level of 130 mg/dL or higher [1]. Hyperlipidemia occurs with total cholesterol in the blood greater than 200 mg/dL [4] In 2006, 51.3% of adults with DM were also taking a statin medication to control cholesterol levels [4]. Statins are the guideline-preferred first-line medication for reducing low-density lipoprotein (LDL) cholesterol [5]. Lifestyle modifications are recommended and consist of improving diet and nutritional intake, weight loss, and physical activity. Lifestyle modifications are recommended in conjunction with primary preventative methods. This is often accomplished by adding a moderate or high-intensity statin medication. Moderate-intensity statin therapy is recommended in those 40–75 years old, whereas high-intensity may be necessary if a patient has a greater risk of atherosclerotic cardiovascular disease. Secondary prevention with the addition of other non-statin cholesterol medications may be considered if there is not adequate control with statin therapy and lifestyle modifications alone [5], with a goal of achieving LDL cholesterol ≤ 55 mg/dL, > 50% reduction in LDL cholesterol, or non-HDL cholesterol ≥ 85 mg/dL [6].

Statin medications work by acting as competitive inhibitors of Hydroxymethylglutaryl (HMG) CoA reductase in the biosynthesis of cholesterol. Statin medications do have pleiotropic effects that could potentially influence DFU healing [7–10]. By selectively competing and interfering with cholesterol biosynthesis, a subsequent decrease in LDL cholesterol, increase in HDL cholesterol, and a decrease in total cholesterol and triglycerides are seen [11]. The mechanism of statin therapy on DFU healing is unclear, but Gulcan et al. proposed that it causes increased vasodilation by increasing nitric oxide synthesis, decreasing endothelin-1 synthesis, and a decrease in vasoconstriction in the lower extremity by reducing available angiotensin-2 [7]. Goggi et al. proposed that neovascularization is promoted by increasing endothelial progenitor cells by increasing vascular endothelial growth factor and capillary density [8]. Statin medications can also be used for aggressive cardiovascular risk management. Aggressive management has been shown to improve mortality rates. However, the effect that statins have on this improvement is unclear [9]. Another theory put forth by Spampinato et al. asserts there is the possibility that statins inhibit farnesyl pyrophosphate and cortisol, which act to inhibit keratinocyte migration and epithelialization [10]. Despite these proposed mechanisms, the relationship between statin use and DFU healing remains unclear.

Multiple studies have evaluated the effects of statin medications on DFU healing [7–10, 12–18]. Fox et al. compared several medication classes and reported an association between DFU wound reduction and intervention for hyperlipidemia via statin therapy. However, this did not reach statistical significance (*p* = 0.057) [12]. Johansen et al. compared dosage of atorvastatin and reported a protective effect from the higher dosage as it related to recurrence of a DFU [13]. Nassaji et al. investigated the relationship of statin use and diabetic foot infection (DFI) outcomes. Those patients without a history of DFI were more likely to be statin users, showing a possible preventative component [14]. Microvascular and macrovascular complications are large contributors to DFU. Nielsen and Nordestgaard investigated if statin use worsened microvascular circulation. Statin users were noted to have lower incidence of diabetic neuropathy, nephropathy, and gangrene of the foot, showing no negative effect on microvasculature [15]. Antonoglou et al. found that statins were protective against macrovascular disease, particularly in delaying progression in those patients with a history of peripheral vascular disease. No clear association was noted with DFU healing and amputation rates [16]. Sohn et al. investigated the incidence of major lower extremity amputation and noted a 35% amputation decrease when compared to no medication or non-statin cholesterol lowering medications [17]. Topical statin medication has also been used to investigate wound healing. This was investigated by Toker et al., who found that topical 1% atorvastatin and 5% atorvastatin had improved wound healing among rats with induced diabetes at 7 and 14 days compared to the control groups [18].

Given these potential mechanisms and prior studies examining the relationship between statin use and aspects of DFU healing, we sought to determine if there was a correlation between DFU healing rates and concomitant statin use. We hypothesized statin therapy promotes DFU healing. Thus, the primary objective of this study was to investigate if there was improved DFU healing among patients on a statin medication compared to those with a DFU who were not taking a statin medication. Secondary outcomes assessed were correlations with wound healing or statin use on data obtained from the retrospective chart review.

## Methods

A retrospective chart review was performed to obtain patient information through University of Michigan Health, a large tertiary care academic health system. A software medical record extraction tool (Data-Direct) was used to identify the study cohorts. Data-Direct is a tool developed by the University of Michigan to assist with data extraction from the unified electronic medical record (EMR). Institutional Review Board approval was obtained (IRB no. HUM-0020797).

We included adult patients with DM with active DFU who were administered a statin medication of either atorvastatin, simvastatin, rosuvastatin, fluvastatin, pravastatin, lovastatin, and pitavastatin between January 2015 and December 2019. Patient encounters were limited to the outpatient setting. Data Direct was used to obtain International Classification of Disease (ICD) versions 9 and 10 diagnostic codes relating to DM, foot ulcer, skin breakdown, infection, and foot and lower extremity. A full list of the included codes can be seen in Appendix A. Common Procedural Terminology (CPT) codes for wound debridement (11,042—11,047) for patients with a DFU were used to assist in evaluating wound progression during usual DFU care.

### Outcome measures

Demographic and comorbid conditions were obtained from the EMR. Information collected included age, sex, race, body-mass index (BMI), type and duration of DM, duration of smoking in pack-years, history of DFU, history of minor lower extremity amputation (e.g., distal to the ankle). Comorbid conditions included hypertension (HTN), coronary artery disease (CAD), hyperlipidemia (HLD), chronic kidney disease (CKD) and stage based on race-based estimated glomerular filtration rate (eGFR) [19], congestive heart failure (CHF), and diabetic retinopathy (DR). Lab values were reviewed and included if they were obtained within 6 months of the onset of the DFU. These included HDL cholesterol, LDL cholesterol, non-HDL cholesterol, total cholesterol, triglyceride, blood glucose levels, hemoglobin A1c, eGFR, creatinine, and urine microalbumin. C-reactive protein (CRP) and erythrocyte sedimentation rate (ESR) starting values were included if obtained at time of onset of DFU and at time of healing of DFU or after 12 weeks of failed healing. CRP and ESR can both assist in diagnosing osteomyelitis and are often obtained when there is concern of an infected DFU, with CRP of 7.9 mg/dL and an ESR of 60 mm/h being suggested thresholds for diagnosing osteomyelitis [20]. Healing was defined as full epithelialization of the DFU with no further drainage as seen at follow-up visits.

Physical examination characteristics at time of DFU onset were reviewed and included palpable pedal pulses, ankle-brachial index (ABI) and toe-brachial index (TBI) values (included if within 6-months of DFU onset), most distal level of intact sensation as tested via 5.07-10 g Semmes Weinstein monofilament [21], location of DFU, and local signs of infection on presentation. Offloading status was recorded to ensure appropriate DFU treatment. Appropriate DFU treatment included regular wound debridement, assessing and addressing vascular status if indicated, infection control, and offloading. The type of statin medication was recorded and included atorvastatin, simvastatin, rosuvastatin, fluvastatin, pravastatin, lovastatin, and pitavastatin. All patients were then followed for 12 weeks to evaluate for healing. We excluded those patients with missing data only from the specific missing variable being calculated.

### Data analysis

Demographic and comorbid data was analyzed using chi square (Χ^2^). Pearson correlation coefficients were obtained for (dichotomous) categorical variables including wound healing and statin use. Strength of correlation was defined as weak for an r-value less than 0.3, mild for an r-value between 0.3–0.6, and strong above 0.6. Statistical analysis was assisted by GraphPad by Dotmatics [22]. All p-values were two-sided, and findings were considered statistically significant at *p* < 0.05.

## Results

There were 109 patients included in the study. Statin users with a DFU (statin cohort) comprised 75 patients and non-statin users with a DFU (non-statin cohort) comprised 34 patients.

### Demographics

Statin users were older, with the average (SD) age being 59.8 (11.4) years versus 52.6 [16] years in the non-statin cohort (*p* < 0.05). Statin users were likely to have had DM for less than 5 years (*p* < 0.05). Supplementary Table 1 demonstrates patient demographic data. Both cohorts were similar in terms of sex, race, BMI, type of DM, smoking history, personal history of DFU, and personal history of lower extremity amputation.

### Comorbidities and physical exam

Statin users had a higher prevalence of HTN (92.0% vs. 67.6%), CAD (36.0% vs. 8.8%), HLD (86.7% vs. 20.6%), CKD status overall (41.3% vs. 17.6%), and CHF (18.7% vs. 2.9%) as compared to non-statin users (all *p* < 0.05). Additionally, CKD stage 3b (20.0% vs. 0%) was higher in statin users (*p *< 0.05). Both cohorts had similar rates of DR. Supplementary Table 2 provides full comorbid condition data of both cohorts as seen below.

Patient physical examination findings to assess peripheral vascular status, including rates of palpable pulses, ABI, and TBI, along with DFU location, were similar amongst groups. Full patient physical examination data for both cohorts can be found in Supplementary Table 3.

### Laboratory values

For statin users and non-statin users, the mean LDL cholesterol was 74.8 mg/dL and 101.5 mg/dL, respectively (*p* < 0.05). For statin users and non-statin users, mean total cholesterol was 151.0 mg/dL and 180.2 mg/dL, respectively (*p* < 0.05). The mean eGFR was 54.0 ml/min/1.73m2 among statin users and 59.0 ml/min among non-statin users (*p* < 0.05). Statin users had a mean creatinine of 1.2 mg/dL and a mean of 0.9 mg/dL in non-statin users (*p* < 0.05). The patient laboratory value data for both cohorts can be found in Supplementary Table 4.

### Statin medication

There were 68% (51/75) of patients taking atorvastatin, 14.7% (11/75) of patients taking simvastatin, 12% (9/75) of patients taking rosuvastatin, 2.7% (2/75) of patients taking pravastatin, and 2.7% (2/75) of patients taking lovastatin, as shown in Supplementary Table 5. No patients were taking fluvastatin or pitavastatin.

### Clinical outcomes

Among statin users, 48.0% (36/75) of patients healed the DFU within 12 weeks. Among non-statin users, 44.1% (15/34) healed the DFU within 12 weeks and showed no correlation (*p* = 0.7).

A significant negative correlation was noted for wound healing and prior minor amputation status with a r-value of -0.3 (*p* < 0.001). Full data for wound healing correlation testing can be seen in Supplementary Table 6.

Separately, significant correlations were noted between statin use and age, duration of DM, LDL cholesterol level, total cholesterol level, HTN, CAD, and HLD with r-values of -0.3 (*p* < 0.01), -0.3 (*p* = 0.001), 0.3 (*p* = 0.01), 0.3 (*p* = 0.03), 0.3 (*p* = 0.001), 0.3 (*p* = 0.003), and 0.7 (*p* < 0.0001), respectively. There was no correlation between wound healing and statin use (*r* = 0.04, *p* = 0.7). Full data for statin use correlation testing can be seen in Supplementary Table 7.

## Discussion

The primary objective of this study was to investigate if there were improved healing rates of DFU among those patients on a statin medication compared to those with DFU who were not taking a statin medication at 12 weeks. Among statin users in our study, there was a 48.0% DFU healing rate and 44.1% DFU healing rate for non-statin users (*p* = 0.7) at 12 weeks. Correlation testing was performed for wound healing and statin use and no correlation was found, with an r-value of 0.04 (*p* = 0.7). These findings did not support our hypothesis that statin use would lead to increased healing rates of DFU.

Margolis et al. found that approximately 24% of patients receiving standard DFU treatment are expected to heal at 12 weeks [23]. Thus, we aimed to evaluate DFU healing within this time frame. In our study, there were improved healing rates for both cohorts compared to 24% seen by Margolis et al. receiving standard treatment at the 12-week mark. Johansen et al. studied two groups taking atorvastatin, with group 1 using the dose of 10 mg and group 2 of 80 mg. In group 1, there was a 100% DFU healing rate and group 2 had a 66% DFU healing rate. Our study did not consider the dosages of statin medications or monitor for recurrence and was retrospective in nature, which could have contributed to the difference in findings. Like Johansen et al., we found no correlation between statin use and DFU healing [13]. Fox et al. assessed DFU size reduction over 6 weeks among patients taking a variety of medications, including statin medications. No DFU size reduction association was noted, but there was a non-significant association for statin use and DFU size reduction compared to other medications. Our study assessed DFU healing instead of DFU size reduction. Similar to Fox et al., no correlation was noted [12].

Among those patients that were statin users, they were likely to have more comorbidities. Statin users were older with a higher comorbid burden at baseline, including higher prevalence of HTN, CAD, HLD, CKD, and CHF. Unsurprisingly, statin users had improved LDL (74.8 mg/dL vs. 101.5 mg/dL, *p* < 0.05) and total cholesterol levels (151.0 mg/dL vs. 180.2 mg/dL, *p* < 0.05). Non-statin users were more likely to have better baseline renal function as noted in eGFR and creatinine levels.

There was a significant negative correlation between prior minor amputations as it relates to DFU healing. As minor amputations are often precipitated by DFU, this finding can be unfortunately common given DFU recurrence rates can be up to 40% after 1 year [24].

There were limitations to this study. First, the study was retrospective, which inherently raises concern for selection bias. We attempted to mitigate this limitation by selecting patients consecutively who met all eligibility requirements. Second, multiple providers with different backgrounds provided DFU care at our institution. This does introduce treatment heterogeneity that would not have occurred with fewer providers. We feel this reflects a real-world scenario as patients are frequently evaluated and treated in this manner. Third, there was variation in the types of DFU that were included, as some were first-time DFU or recurrences. On DFU that healed, there was no follow-up period to ensure they remained healed. With the high DFU recurrence rate after a healing event, this could be studied further [24]. We attempted to address this by selecting a DFU at random during the study period but did not differentiate between first-time or recurrent DFU. Fourth, there was no distinction on low, moderate, or high-intensity statin therapy as dosages were not recorded. Our study did not find a difference in healing rates between our evaluated cohorts. One of the main studies which evaluated the healing effect of statin medications and DFU healing specifically questioned if statin dose affected DFU healing and recurrence rates [13]. The intensity of statin therapy could be an avenue for further research.

## Conclusions

Based on our study, there was not a correlation between DFU healing and statin therapy. Future studies should include multiple institutions to prospectively study various dosages of statin medication, topical statin therapy, prior amputations and DFU healing, and pre-specify if the DFU in question is new or recurrent.

### Supplementary Information

**Supplementary Material 1. **

## Data Availability

The datasets used and/or analyzed during the current study are available from the corresponding author on reasonable request.

## Abbreviations

DM: Diabetes mellitus
DFU: Diabetic foot ulcer
HDL: High-density lipoprotein
LDL: Low-density lipoprotein
HMG: Hydroxymethylglutaryl
DFI: Diabetic foot infection
EMR: Electronic medical record
ICD: International Classification of Disease
CPT: Common Procedural Terminology
BMI: Body-mass index
HTN: Hypertension
CAD: Coronary artery disease
HLD: Hyperlipidemia
CKD: Chronic kidney disease
eGFR: Estimated glomerular filtration rate
CHF: Congestive heart failure
DR: Diabetic retinopathy
CRP: C-reactive protein
ESR: Erythrocyte sedimentation rate
ABI: Ankle-brachial index
TBI: Toe-brachial index
