# Supplementary material for: The impact of statin therapy on the healing of diabetic foot ulcers: a case–control series
Source: Clin Diabetes Endocrinol. 2024 Jul 10;10:19. doi: 10.1186/s40842-024-00175-8 (PMC11234678; doi:10.1186/s40842-024-00175-8)
Supplement: Supplementary file 1 — Supplementary Material 1. [file 40842_2024_175_MOESM1_ESM.docx]

**Supplementary Material**

**The Impact of Statin Therapy on the Healing of Diabetic Foot Ulcers: A Case-Control Series.**

**O’Dell, Brennen DPM^1^*; Rothenberg, Gary DPM^1^; Holmes, Crystal DPM^1^; Priesand, Sari DPM^1^; Mizokami-Stout, Kara MD^1^; Brandt, Eric J. MD^2, 3^; Schmidt, Brian M. DPM^1^**

*** Correspondence:** Brennen O’Dell: brenneno@med.umich.edu

^1^Department of Internal Medicine, Division of Metabolism, Endocrinology, and Diabetes, University of Michigan Medical School, Ann Arbor, Michigan, United States

^2^Department of Internal Medicine, Division of Cardiovascular Medicine, University of Michigan Medical School, Ann Arbor, Michigan, United States

^3^Institute for Healthcare Policy, University of Michigan Medical School, Ann Arbor, Michigan, United States

**Supplementary Data**

**Appendix**

Appendix A List of ICD-9 and ICD-10 diagnostic codes included for data collection

- DM: ICD-9 249.80, 250.60, 250.61, 250.70, 250.80, 250.81, 250.82, 713.5, V58.67; ICD-10 E10.610, E11.610, E11.65, E11.69, Z79.4
- Foot ulcer: ICD-9 707.9, 707.10, 707.14, 707.15; ICD-10 E08.621, E08.622, E09.621, E09.622, E10.621, E10.622, E11.621, E11.622, E13.621, E13.622, L97.402, L97.409, L97.411, L97.412, L97.413, L97.414, L97.416, L97.418, L97.419, L97.421, L97.422, L97.423, L97.424, L97.425, L97.426, L97.429, L97.509, L97.511, L97.512, L97.513, L97.514, L97.516, L97.519, L97.521, L97.522, L97.523, L97.524, L97.526, L97.529
- Skin breakdown: ICD-9 785.4; ICD-10 E08.620 E08.628. E09.620, E09.628, E10.620, E10.628, E11.620, E11.628, E13.620, E13.628
- Infection: ICD-9 686.9; ICD-10 L08.9
- Foot and lower extremity: ICD-10 E11.52

**Supplementary Tables**

**Table 1 Demographic characteristics for statin users and non-statin users**

| Characteristics | Total (n=109) | Statin (n=75) | No statin (n=34) | P-value |
| --- | --- | --- | --- | --- |
| Age, years, mean ± SD | 57.0 ± 14.3 | 59.8 ± 11.4 | 52.6 ± 16 | **< 0.01*** |
| Sex, % Men (n) | 67.0 (73) | 72 (54) | 55.9 (19) | 0.1 |
| Race |  |  |  |  |
| Caucasian, % (n) | 80.7 (88) | 82.7 (62) | 76.5 (26) | 0.5 |
| Black, % (n) | 14.7 (16) | 16 (12) | 11.8 (4) | 0.6 |
| Hispanic, % (n) | 0.9 (1) | 0 (0) | 2.9 (1) | 0.14 |
| Asian, % (n) | 1.8 (2) | 1.3 (1) | 2.9 (1) | 0.6 |
| American Indian, % (n) | 0.9 (1) | 0 (0) | 2.9 (1) | 0.1 |
| Pacific Islander, % (n) | 0 (0) | 0 (0) | 0 (0) | 0 |
| Other, % (n) | 0.9 (1) | 0 (0) | 2.9 (1) | 0.1 |
| BMI |  |  |  |  |
| Below 18.5, % (n) | 0 (0) | 0 (0) | 0 (0) | 0 |
| 18.5 - 24.9, % (n) | 11 (12) | 9.3 (7) | 14.7 (5) | 0.4 |
| 25.0 - 29.9, % (n) | 27.5 (30) | 32 (24) | 17.6 (6) | 0.1 |
| 30.0 - 34.9, % (n) | 25.7 (28) | 22.7 (17) | 32.4 (11) | 0.3 |
| 35.0 - 39.9, % (n) | 24.8 (27) | 26.7 (20) | 20.6 (7) | 0.5 |
| > 40, % (n) | 11 (12) | 9.3 (7) | 14.7 (5) | 0.4 |
| DM type, % Type 2 (n) | 78.9 (86) | 80 (60) | 76.5 (26) | 0.7 |
| Duration of DM |  |  |  |  |
| Unknown, % (n) | 13.8 (15) | 8 (6) | 26.5 (9) | **< 0.01*** |
| < 5 years, % (n) | 14.7 (16) | 9.3 (7) | 26.5 (9) | **< 0.05*** |
| 5-10 years, % (n) | 11.9 (13) | 14.7 (11) | 5.9 (2) | 0.2 |
| 11-15 years, % (n) | 16.5 (18) | 18.7 (14) | 11.8 (4) | 0.4 |
| 16-20 years, % (n) | 12.8 (14) | 13.3 (10) | 11.8 (4) | 0.8 |
| > 20 years, % (n) | 30.3 (33) | 36 (27) | 17.6 (6) | 0.05 |
| Smoking (pack years) |  |  |  |  |
| 0, % (n) | 46.8 (51) | 49.3 (37) | 41.2 (14) | 0.4 |
| 0-15, % (n) | 25.7 (28) | 17.3 (13) | 44.1 (15) | **< 0.01*** |
| 16-30, % (n) | 8.3 (9) | 9.3 (7) | 5.9 (2) | 0.5 |
| 31-45, % (n) | 5.5 (6) | 8 (6) | 0 (0) | 0.09 |
| > 46, % (n) | 13.8 (15) | 16 (12) | 8.8 (3) | 0.3 |
| Prior DFU, % (n) | 70.6 (77) | 73.3 (55) | 64.7 (22) | 0.4 |
| Prior amputation, % (n) | 30.3 (33) | 33.3 (25) | 23.5 (8) | 0.3 |

Legend:

* Statistically significant

Abbreviations: SD: Standard deviation; BMI: Body Mass Index; DM: Diabetes Mellitus; DFU: Diabetic Foot Ulcer

**Table 2 Comorbid conditions for statin users and non-statin users**

| Co-morbid condition | Overall % (n) | Statin % (n=75) | No statin % (n=34) | p-value |
| --- | --- | --- | --- | --- |
| HTN | 84.4 (92) | 92 (69) | 67.6 (23) | **< 0.01*** |
| CAD | 27.5 (30) | 36 (27) | 8.8 (3) | **< 0.01*** |
| HLD | 66.1 (72) | 86.7 (65) | 20.6 (7) | **< 0.01*** |
| CKD | 33.9 (37) | 41.3 (31) | 17.6 (6) | **< 0.05*** |
| 3a | 21.1 (23) | 22.7 (17) | 17.6 (6) | 0.6 |
| 3b | 11 (12) | 20 (12) | 0 (0) | **< 0.05*** |
| 5 | 1.8 (2) | 2.7 (2) | 0 (0) | 0.3 |
| CHF | 13.8 (15) | 18.7 (14) | 2.9 (1) | **< 0.05*** |
| DR | 32.1 (35) | 34.7 (26) | 26.4 (9) | 0.4 |

Legend:

* Statistically significant

Abbreviations: HTN: Hypertension; CAD: Coronary Artery Disease; HLD: Hyperlipidemia; CKD: Chronic Kidney Disease; CHF: Congestive Heart Failure; DR: Diabetic Retinopathy

**Table 3 Physical examination characteristics for statin users and non-statin users**

| Examination | Total (n=109) | Statin (n=75) | No statin (n=34) | P-value |
| --- | --- | --- | --- | --- |
| Palpable pedal pulses, % (n) | 83.5 (91) | 80 (60) | 91.2 (31) | 0.2 |
| ABI, % (n) |  |  |  |  |
| > 1.30, % (n) | 15.6 (17) | 17.3 (13) | 11.8 (4) | 0.5 |
| 0.91 - 1.30, % (n) | 37.6 (41) | 36 (27) | 41.2 (14) | 0.6 |
| 0.71 - 0.90, % (n) | 4.6 (5) | 5.3 (4) | 2.9 (1) | 0.6 |
| 0.41 - 0.70, % (n) | 6.4 (7) | 8 (6) | 2.9 (1) | 0.3 |
| < 0.40, % (n) | 1.8 (2) | 2.7 (2) | 0 (0) | 0.3 |
| TBI, % (n) |  |  |  |  |
| Non-compressible, % (n) | 2.8 (3) | 1.3 (1) | 5.9 (2) | 0.2 |
| > 0.70, % (n) | 43.1 (47) | 42.7 (32) | 44.1 (15) | 0.9 |
| 0.51 - 0.70, % (n) | 6.4 (7) | 6.7 (5) | 5.9 (2) | 0.9 |
| 0.35 - 0.50, % (n) | 3.7 (4) | 5.3 (4) | 0 (0) | 0.2 |
| < 0.35, % (n) | 10.1 (11) | 13.3 (10) | 2.9 (1) | 0.1 |
| DPN level, % (n) |  |  |  |  |
| None, % (n) | 4.6 (5) | 2.7 (2) | 8.6 (3) | 0.2 |
| Toes, % (n) | 21.2 (23) | 25.3 (19) | 11.4 (4) | 0.1 |
| Midfoot, % (n) | 18.4 (20) | 21.3 (16) | 11.4 (4) | 0.2 |
| Rearfoot, % (n) | 11.9 (13) | 13.3 (10) | 8.8 (3) | 0.5 |
| Lower leg, % (n) | 43.1 (47) | 37.3 (28) | 55.9 (19) | 0.07 |
| Offloading, % (n) | 96.3 (105) | 96 (72) | 97.1 (33) | 0.8 |
| Location, % (n) |  |  |  |  |
| Toes, % (n) | 43.1 (47) | 44 (33) | 41.2 (14) | 0.8 |
| Forefoot, % (n) | 34.9 (38) | 36 (27) | 32.4 (11) | 0.7 |
| Midfoot, % (n) | 14.7 (16) | 14.7 (11) | 14.7 (5) | 1 |
| Rearfoot, % (n) | 7.3 (8) | 5.3 (4) | 11.8 (4) | 0.2 |
| Signs of infection on presentation, % (n) | 30.3 (33) | 32 (24) | 26.5 (9) | 0.6 |

Legend:

Abbreviations: ABI: Ankle-Brachial Index; TBI: Toe-Brachial Index; DPN: Diabetic Peripheral Neuropathy

**Table 4 Laboratory values for statin users and non-statin users**

| Lab values | Total (n=109) | Statin (n=75) | No statin (n=34) | P-value |
| --- | --- | --- | --- | --- |
| HDL (mmol/L) | 44.6 ± 16.7 | 43 ± 16.6 | 50.2 ± 16.7 | 0.2 |
| Non-HDL (mmol/L) | 111.0 ± 40.5 | 108 ± 38.3 | 129.9 ± 33.1 | 0.07 |
| LDL (mmol/L) | 80.8 ± 33.9 | 74.8 ± 33.7 | 101.5 ± 28.4 | **< 0.05*** |
| Total cholesterol (mmol/L) | 157.5 ± 43.2 | 151 ± 43.9 | 180.2 ± 34.9 | **< 0.05*** |
| Triglyceride (mmol/L) | 184.7 ± 223.7 | 193.6 ± 252 | 153.8 ± 87 | 0.6 |
| Blood glucose (mg/dL) | 192.0 ± 96.5 | 201.6 ± 96.8 | 167 ± 94.6 | 0.1 |
| Hemoglobin A1c (%) | 8.5 ± 2.2 | 8.6 ± 2 | 8.2 ± 2.5 | 0.4 |
| CRP start (mg/dL) | 3.3 ± 5.2 | 4 ± 5.9 | 1.9 ± 3.8 | 0.2 |
| CRP end (mg/ dL) | 4.4 ± 7.4 | 4.5 ± 7.9 | 4.1 ± 7.3 | 0.9 |
| ESR start (mm) | 36.3 ± 24.0 | 39.3 ± 26.7 | 29.6 ± 16.5 | 0.2 |
| ESR end (mm) | 40.5 ± 27.3 | 41.5 ± 30.6 | 38.1 ± 22.6 | 0.8 |
| eGFR (mL/min/1.73m2) | 55.4 ± 9.5 | 54 ± 10.8 | 59 ± 2.8 | **< 0.05*** |
| Creatinine (mg/dL) | 1.1 ± 0.7 | 1.2 ± 0.7 | 0.9 ± 0.2 | **< 0.05*** |
| Urine microalbumin (ug,mL) | 167.7 ± 415.9 | 206 ± 479.9 | 52.8 ± 84.1 | 0.3 |

Legend:

* Statistically significant

Presented as mean ± SD

Abbreviations: SD: Standard Deviation; HDL: High-Density Lipoprotein; LDL: Low-Density Lipoprotein; CRP: C-Reactive Protein; ESR: Erythrocyte Sedimentation Rate; eGFR: Estimated Glomerular Filtration Rate

**Table 5 Statin medication**

| Statin Medication | Statin % (n=75) |
| --- | --- |
| Atorvastatin | 68.0 (51) |
| Simvastatin | 14.7 (11) |
| Rosuvastatin | 12.0 (9) |
| Pravastatin | 2.7 (2) |
| Lovastatin | 2.7 (2) |

**Table 6 Wound healing correlation testing**

| Variables | Wound healing r-value | Wound healing p-value |
| --- | --- | --- |
| Age | 0.006 | 0.9 |
| Sex | 0.07 | 0.5 |
| Race | -0.07 | 0.5 |
| BMI (kg/m2) | -0.08 | 0.4 |
| DM type | -0.1 | 0.2 |
| Duration of DM | 0.04 | 0.7 |
| Smoking (pack years) | 0.07 | 0.5 |
| Statin | 0.04 | 0.7 |
| Statin medication | 0.1 | 0.5 |
| Non-statin medication | -0.05 | 0.6 |
| HDL | 0.2 | 0.3 |
| LDL | -0.002 | 0.8 |
| Total cholesterol | 0.009 | 0.6 |
| Triglyceride | 0.03 | 0.8 |
| Blood glucose | -0.07 | 0.5 |
| Hemoglobin A1c | 0.2 | 0.1 |
| Creatinine | 0.09 | 0.3 |
| eGFR | -0.08 | 0.4 |
| HTN | 0.05 | 0.6 |
| CAD | -0.08 | 0.4 |
| HLD | 0.01 | 0.9 |
| CKD | -0.09 | 0.4 |
| CKD 3a | -0.1 | 0.2 |
| CKD 3b | 0.08 | 0.4 |
| CKD 5 | -0.1 | 0.2 |
| CHF | -0.5 | 0.6 |
| DR | 0.1 | 0.1 |
| DPN level | 0.2 | 0.02 |
| Palpable pulses | 0.07 | 0.5 |
| Prior DFU | -0.2 | 0.09 |
| Prior amputation | **-0.3** | **0.0003*** |
| Offloading | -0.007 | 0.9 |
| Location | 0.04 | 0.7 |
| Local SOI | 0.06 | 0.5 |
| Surface area at presentation | -0.003 | 0.9 |

Legend:

* Statistically significant

Abbreviations: BMI: Body Mass Index; DM: Diabetes Mellitus; HDL: High-Density; LDL: Low-Density Lipoprotein; CRP: C-Reactive Protein; ESR: Erythrocyte Sedimentation Rate; eGFR: Estimated Glomerular Filtration Rate; HTN: Hypertension; CAD: Coronary Artery Disease; HLD: Hyperlipidemia; CKD: Chronic Kidney Disease; CHF: Congestive Heart Failure; DR: Diabetic Retinopathy; DPN: Diabetic Peripheral Neuropathy; PAD: Peripheral Arterial Disease; DFU: Diabetic Foot Ulceration; SOI: Signs of infection

**Table 7 Statin use correlation testing**

| Variables | Statin use r-value | Statin use p-value |
| --- | --- | --- |
| Age | **-0.3** | **0.008*** |
| Sex | 0.2 | 0.1 |
| Race | 0.2 | 0.04 |
| BMI (kg/m2) | 0.03 | 0.7 |
| DM type | -0.04 | 0.7 |
| Duration of DM | **-0.3** | **0.001*** |
| Smoking (pack years) | -0.1 | 0.3 |
| Non-statin medication | 0.1 | 0.2 |
| HDL | 0.2 | 0.2 |
| LDL | **0.3** | **0.01*** |
| Total cholesterol | **0.3** | **0.03*** |
| Triglyceride | -0.07 | 0.6 |
| Blood glucose | -0.2 | 0.1 |
| Hemoglobin A1c | -0.09 | 0.4 |
| Creatinine | -0.2 | 0.03 |
| eGFR | 0.2 | 0.02 |
| HTN | **0.3** | **0.001*** |
| CAD | **0.3** | **0.003*** |
| HLD | **0.7** | **< 0.0001*** |
| CKD | 0.2 | 0.02 |
| CKD 3a | 0.06 | 0.6 |
| CKD 3b | 0.2 | 0.01 |
| CKD 5 | 0.09 | 0.3 |
| CHF | 0.2 | 0.03 |
| DR | 0.08 | 0.4 |
| DPN level | 0.1 | 0.2 |
| Palpable pulses | -0.1 | 0.2 |
| Prior DFU | 0.09 | 0.4 |
| Prior amputation | 0.1 | 0.3 |
| Offloading | -0.09 | 0.4 |
| Location | 0.08 | 0.4 |
| Local SOI | 0.06 | 0.6 |
| Surface area at presentation | 0.04 | 0.7 |
| Amputation | 0.1 | 0.3 |
| Healed | 0.04 | 0.7 |

Legend:

* Statistically significant

Abbreviations: BMI: Body Mass Index; DM: Diabetes Mellitus; HDL: High-Density; LDL: Low-Density Lipoprotein; CRP: C-Reactive Protein; ESR: Erythrocyte Sedimentation Rate; eGFR: Estimated Glomerular Filtration Rate; HTN: Hypertension; CAD: Coronary Artery Disease; HLD: Hyperlipidemia; CKD: Chronic Kidney Disease; CHF: Congestive Heart Failure; DR: Diabetic Retinopathy; DPN: Diabetic Peripheral Neuropathy; PAD: Peripheral Arterial Disease; DFU: Diabetic Foot Ulceration; SOI: Signs of infection
